# A laboratory framework for ongoing optimization of amplification-based genomic surveillance programs

**DOI:** 10.1128/spectrum.02202-23

**Published:** 2023-11-15

**Authors:** Connie Lam, Jessica Johnson-Mackinnon, Kerri Basile, Winkie Fong, Carl J.E. Suster, Mailie Gall, Jessica Agius, Shona Chandra, Jenny Draper, Elena Martinez, Alexander Drew, Qinning Wang, Sharon C. Chen, Jen Kok, Dominic E. Dwyer, Vitali Sintchenko, Rebecca J. Rockett

**Affiliations:** 1 Centre for Infectious Diseases and Microbiology - Public Health, Institute for Clinical Pathology and Medical Research Westmead Hospital, Westmead, Australia; 2 Faculty of Medicine and Health, Sydney Infectious Diseases Institute, The University of Sydney, Sydney, New South Wales, Australia; 3 Centre for Infectious Diseases and Microbiology - Laboratory Services, Institute for Clinical Pathology and Medical Research, NSW Health Pathology, Sydney, Australia; 4 Faculty of Medicine and Health, School of Medical Sciences, The University of Sydney, Sydney, New South Wales, Australia; Nevada State Public Health Laboratory, Reno, Nevada, USA

**Keywords:** genomic surveillance, whole genome sequencing, public health, viral sequencing

## Abstract

Constantly evolving viral populations affect the specificity of primers and the quality of genomic surveillance. This study presents a framework for the continuous optimization of sequencing efficiency for public health surveillance based on the ongoing evolution of the COVID-19 pandemic. SARS-CoV-2 genomic clustering capacity based on three amplification-based whole genome sequencing schemes was assessed using decreasing thresholds of genome coverage and measured against epidemiologically linked cases. The overall genome coverage depth and individual amplicon depth were used to calculate an amplification efficiency metric. Significant loss of genome coverage over time was documented, which was recovered by optimization of primer pooling or implementation of new primer sets. A minimum of 95% genome coverage was required to cluster 94% of epidemiologically defined SARS-CoV-2 transmission events. Clustering resolution fell to 70% when only 85% genome coverage was achieved. The framework presented in this study can provide public health genomic surveillance programs with a systematic process to ensure an agile and effective laboratory response during rapidly evolving viral outbreaks.

**IMPORTANCE:**

This study provides a laboratory framework to ensure ongoing relevance and performance of amplification-based whole genome sequencing to strengthen public health surveillance during extended outbreaks or pandemics. The framework integrates regular reviews of the performance of a genomic surveillance system and highlights the importance of ongoing monitoring and the identification and implementation of improvements to whole genome sequencing methods to enhance public health responses to pathogen outbreaks.

## INTRODUCTION

The near-real-time generation and sharing of SARS-CoV-2 genomes has enabled unprecedented international surveillance of SARS-CoV-2 evolution (1). SARS-CoV-2 whole genome sequencing (WGS) has been enabled by the use of multiplex polymerase chain reaction (PCR) amplification to selectively amplify SARS-CoV-2 from clinical specimens. Several tiling primer schemes were rapidly designed after the identification of the novel coronavirus causing COVID-19 disease in January 2020 (2 – 5). These methods have been employed at scale during large outbreaks and increasing case numbers. At the start of the COVID-19 pandemic, our laboratory initially implemented a long-tiled amplicon WGS protocol (5), which produced high-quality consensus genomes in a circulating viral population with low levels of mutational changes. However, amplification-based sequencing methods are sensitive to mutations or indels within primer sites, thus resulting in inconsistencies in amplification across the viral genome. The variability (and potential non-amplification) of SARS-CoV-2 genomic regions ultimately affects the overall genome coverage required for in-depth genomic surveillance. Ongoing viral evolution, the emergence of multiple variants of concern (VOCs) over the course of 2021, combined with increased case numbers in New South Wales (NSW), Australia, led to varying reductions in genome coverage and increasing difficulty in providing the level of genomic resolution established at the beginning of the COVID-19 pandemic. For example, the Omicron VOC has accumulated in excess of 53 mutations in comparison to the Wuhan-Hu-1 strain (6) (which the original ARTIC method was designed to survey) and continues to evolve. Over 30 of these mutations are concentrated in the spike receptor-binding domain (RBD) within the coding region of the primary vaccine antigen (7). The inability to amplify and sequence even a small part of the genome, particularly within the RBD, can have substantial effects on the integrity of genomic surveillance programs, including the loss of the ability to reliably detect emerging SARS-CoV-2 lineages (8 – 10). Furthermore, rapid identification of punctuated or saltational evolution may be impacted, leading to unrecognized circulation of variants with potential increased transmissibility, virulence, or capacity to evade vaccine- or infection-induced immunity.

The importance of maintaining high sequencing quality to accurately reconstruct viral evolution has been recognized (11). The ongoing decline in the efficiency of factors associated with amplification-based WGS (e.g., primer specificity and multiplex cross-reactivities) placed additional demands on genomic laboratories to maintain consistent levels of sequencing depth and genome coverage within increasingly diverse viral populations (11). SARS-CoV-2 genomic surveillance has illuminated the need for ongoing assessment and optimization of amplification-based WGS to ensure the sustained quality of data and the agility and usefulness of surveillance programs. This study describes a framework for the optimization of genome sequencing efficiency based on our experience with SARS-CoV-2 surveillance during the COVID-19 pandemic. We quantified genome coverage loss across 3 years of the pandemic using a number of SARS-CoV-2 primer schemes. We established quality metrics for genome coverage and primer efficiency and applied them to trigger protocol reviews and modifications. The validation methodology to optimize multiple primer schemes to improve genome coverage and to sustain high-quality genome sequencing for public health surveillance is also outlined.

## MATERIALS AND METHODS

### Retrospective review of SARS-CoV-2 genome coverage

SARS-CoV-2 genomes sequenced between April 2020 and May 2022 in NSW at the Microbial Genomics Reference Laboratory, Centre for Infectious Diseases and Microbiology Laboratory Services (CIDMLS)*,* Institute for Clinical Pathology and Medical Research (ICPMR) - NSW Health Pathology, Australia, were included in this study. Sequencing was performed as a part of the NSW Health program of integrated public health genomic surveillance, which additionally collected data on epidemiological links between cases. Sequences were mapped to the SARS-CoV-2 reference genome [National Center for Biotechnology Information (NCBI) GenBank accession MN908947.3] and analyzed to determine genome coverage. Only genomes with more than 500,000 mappable reads to the reference genome were included in the retrospective genome coverage review. The genomes were then categorized into VOCs based on Pangolin lineage designation (version 3.0.3) (12, 13).

### Representative viral cultures, RNA extraction, and serial dilutions

Viral cultures representing seven distinct SARS-CoV-2 lineages causing COVID-19 outbreaks in NSW were selected. Each of these seven cultures were grown from clinical samples, and SARS-CoV-2 RNA was detected by reverse transcriptase real-time PCR (RT-PCR) as previously described (14). Total RNA was extracted from 200 µL of culture supernatant using either the QiagenRNeasy Mini Kit (based on the manufacturer’s instructions) or the automated Roche MagNA Pure 96 instrument together with the MagNA Pure 96 DNA and Viral NA Small Volume Kit. cDNA was generated for all samples using the LunaScript RT SuperMix Kit (New England BioLabs). A sufficient volume was prepared to perform serial dilutions (10^−2^ to 10 ^−7^) of the cDNA from each culture. RT-PCR targeting the SARS-CoV-2 N-gene was then performed for each sample dilution to measure the SARS-CoV-2 load as previously described (14).

### Amplification-based WGS

Three previously published amplification-based sequencing protocols, Long Amplicon (5), ARTIC (v3 and v4) (3), and Midnight (v1) (4), and a newly designed protocol, CIDM-PH oMicron (CIDoMi) (see methods below), were used. Long Amplicon and ARTIC methods were prepared as described previously (3, 5, 15).

Midnight v1 and CIDoMi primer sets were prepared following the SARS-CoV-2 1,200-bp protocol (4). Briefly, PCR master mixes were prepared for each pool of primers. Each PCR contained 12.5 µL of Q5 High Fidelity 2× Master Mix (New England Biolabs), 1.1 µL of pooled primer mixes at 10 µM (final concentration of each primer was ~10–11 pM), and 2.5 µL of template and molecular grade water to a final reaction volume of 25 µL. The cycling conditions were as follows: initial denaturation at 98°C for 30 s, followed by 35 cycles of 98°C for 15 s and 65°C for 5 min.

For all amplification protocols, PCR amplicons for pools 1 and 2 were combined for each sample and purified with a 1:1 ratio of AMPureXP beads (Beckman Coulter) and eluted in 30 µL of sterile water. Purified products were quantified using the Qubit 1× dsDNA HS Assay Kit (Thermo Fisher Scientific) and diluted to 1 ng for library preparation. Sequencing libraries were prepared using Nextera XT (Illumina) according to the manufacturer’s instructions. Libraries were then sequenced to generate 75-bp reads on either the Illumina iSeq or MiniSeq platforms.

### Bioinformatic analysis

Raw reads were quality controlled using an in-house pipeline (16). Briefly, demultiplexed reads were quality trimmed using Trimmomatic v0.36 (sliding window of 4, minimum read quality score of 20, leading/trailing quality of 5, and minimum length of 36 after trimming). Reads were mapped to the reference SARS-CoV-2 genome (NCBI GenBank accession MN908947.3) using BWA-mem version 0.7.17, with unmapped reads discarded. Variant calling and consensus genome generation were performed using iVar (v 1.2). The average read depth and genome coverage were calculated using Samtools coverage (v 1.15.1). Genomes with >98% coverage and >100× average read depth were considered high quality and included in further analysis. Read depth per amplicon was calculated using mosdepth (v 0.3.4)

### Determining SARS-CoV-2 amplification efficiency

The mean depth of coverage of the entire genome was determined using Samtools coverage (v 1.15.1). The median depth of each non-overlapping amplicon was calculated separately using mosdepth (v 0.3.4). A sequence read fraction (SRF) was used as a quantitative measure of amplicon efficiency. SRF was calculated for each amplicon of each isolate by dividing the median depth of coverage per amplicon by the average depth of coverage of the entire genome. Amplicons with a median SRF of >1.0 were considered efficient, amplicons with an SRF between 0.05–1.0 were classified as inefficient, and amplicons with a median SRF of <0.05 were designated as critically underperforming in the amplicon pool.

### Optimization of WGS amplification

Underperforming amplicons were optimized by increasing primer concentrations as previously described by the COVID-19 Genomics United Kingdom (COG-UK) Consortium guidelines (17). Critically underperforming amplicons (SRF < 0.05) were identified for three of the WGS amplification methods (ARTIC, Midnight v1, and CIDoMi). The corresponding primer concentrations for these amplicons were increased relative to other primers within their primer pool. Overperforming amplicons (SRF > 1.5) had corresponding primer concentrations reduced within the primer multiplex. Rebalanced primer concentrations for all WGS methods were then tested against the same panel of representative SARS-CoV-2 culture dilutions. Amplicon SRFs were then calculated for each optimized method and compared to standard equimolar pooling.

### Primer design for CIDoMi, a new amplification-based WGS schema

CIDoMi, a new amplification-based WGS method, was designed using PrimalScheme (version 1.3.2) (18). Eight SARS-CoV-2 genomes from samples collected between December 2021 and February 2022 were aligned and used as input into PrimalScheme (Supplemental Material). Genomes represented VOCs: Delta (AY39.1.2 and AY39.1.3), Omicron (BA.1, BA.1.1, BA.1.17, and BA.2), and Lineage B (NCBI genome accession MN908947.3) sequences. Primers were designed to produce 1,200-bp amplicons using default parameters. The resulting primer sequences were evaluated for primer mismatches against an alignment of 274 Omicron genomes circulating in Australia (accessed on gisaid.org 22–03-2022, Supplemental Material).

### Determination of minimum levels of genome coverage for genomic clustering and genotyping

A set of Delta genomes was curated from our routine SARS-CoV-2 genomic surveillance and used to simulate minimum levels of genome coverage required for accurate genomic and epidemiological clustering. Only genomes that had >98% coverage and also had comprehensive epidemiological follow-up data available to support genomic links were included. A total of 159 Delta genomes met these criteria, and the appropriate genomic data and epidemiological metadata for these genomes were curated into a set of samples used in downstream genome coverage simulations. The SRF for each of the amplicons within the 159 genomes was generated using the methods described above, and amplicons were then ranked by SRF from least to highest amplification efficiency. Genome regions corresponding to the lowest 5%, 15%, and 25% SRFs were artificially replaced with N’s in the whole genome alignment using bedtools (v 2.25.0) to represent poor coverage. The newly created masked genomes, now representing 95%, 85%, and 75% genome coverage, respectively, were aligned to the reference genome using MAFFT (v 7.487). Each new set of consensus genomes was clustered using single nucleotide polymorphism (SNP) distances from snp-dists (v 0.6), and consensus trees were drawn for each coverage mask using MAFFT and IQTree (v 1.6.7) as previously described (19). Comparisons between median SRFs for equimolar and rebalanced protocols were performed using two-tailed Mann-Whitney tests.

## RESULTS

### Optimization of primer efficiency of amplification-based WGS protocols

Amplicon efficiency across three WGS amplification methods was tested and optimized using a set of viral cultures representing wild-type SARS-CoV-2 [Lineage A.2.2 (*n* = 1)], as well as WHO-designated VOCs: Beta [Lineage B.1.35 (*n* = 1)], Delta (*n* = 4), and Omicron [BA.1 (*n* = 1) and BA.2 (*n* = 1)]. Across all variants, the median concentration of culture extract dilutions ranged from 1.6 × 10^9^ copies/µL [cycle threshold (Ct) = 24] to 1.6 × 10^2^ copies/µL (Ct = 35) (Fig. S1).

**Fig 1:**
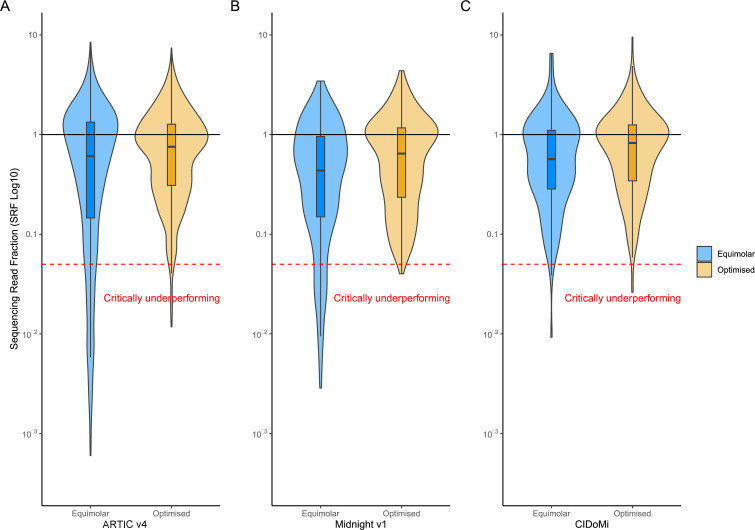
Violin and box plots for (A) ARTIC v4, (**B**) Midnight v1, and (C) CIDoMi SARS-CoV-2 whole genome sequencing (WGS) methods. Blue violin and box plots represent equimolar primer pools, whereas yellow violin and box plots have been calculated from primer pools that have been optimized and rebalanced based on sequence read fractions (SRFs) of amplicons from the ARTIC v3, Midnight v1, and CIDoMi SARS-CoV-2 WGS protocols. The black line (SRF = 1) represents ideal amplicon efficiency, and the red dotted line (i.e., SRF < 0.05) is the threshold for critically underperforming amplicons. Medians for all three optimized WGS protocols are higher than their respective equimolar protocols; however, no significant difference was observed (*P* > 0.1).

A total of 13/24 culture dilutions produced >98% genome coverage using both equimolar and optimized ARTIC v4 primers. As expected, the average Ct value of complete genomes was lower (Ct 29.5, range 25.58–34.84) than that of incomplete genomes (Ct 35.9, range 33.58–38.04). However, equimolar primers demonstrated a broader range of SRF efficiencies, with 15.2% (15/99) of amplicons critically underperforming (SRF < 0.05). These included amplicons predominantly in the ORF1ab region (amplicons 1, 3, 5, 7, 9, 10, 11, 13, 14, 18, 32, 33, 50, and 60) and one that spanned the E and M genes (amplicon 88). Upon primer rebalancing, only one ARTIC v4 amplicon had a median SRF of <0.05 (Fig. 1A). This was amplicon 72 within the spike coding region.

WGS with Midnight v1 primers resulted in 54% (13/24) and 46% (11/24) of complete genomes from equimolar and rebalanced primers, respectively. Two dilutions failed library preparation after amplification with the optimized CIDoMi primers and were not repeated. The average SARS-CoV-2 Ct value of complete genomes was 29.55 (range 25.58–33.94) compared to 35.93 for incomplete genomes (range 32.85–38.22) (Fig. S1). However, 17.2% (5/29) of amplicons were critically underperforming using equimolar primers (SRF <0.05). This included two amplicons in the ORF1ab region (2 and 6), two amplicons in the spike gene (22 and 23), and one spanning the M, ORF6, and ORF7 genes (amplicon 27). After rebalancing primers of critically underperforming Midnight v1 amplicons, all amplicons had a median SRF of >0.05 (Fig. 1B).

### Design and performance of CIDoMI primers

SARS-CoV-2 Omicron (Lineage B.1.1.529) was first identified in South Africa in November 2021 but rapidly replaced Delta as the dominant lineage throughout the world (20, 21). A review of the SRF of 4,039 Omicron genome sequences circulating in Australia during our study period indicated that three Midnight v1 amplicons (amplicons 10, 24, and 28) consistently failed to sufficiently amplify and produce adequate sequencing reads. In addition, 55% (16/29) of Midnight v1 amplicons had a median SRF of <1.0, and three amplicons had an SRF of ≤0.05 (i.e., amplicons 10, 24, and 28). Further investigation identified mutations and indels within primer binding regions and suggested that these amplicons could not be recovered by adjusting primer concentrations alone.

We addressed the loss of these informative regions by designing a new SARS-CoV-2 WGS protocol using PrimalScheme (18), as described in the methods section. Eight Omicron genomes produced by our laboratory between December 2021 and February 2022 were used as representative Omicron sequences. Initial testing of CIDoMi with a dilution series of viral cultures of Omicron BA.1 and BA.2 (Fig. S1) demonstrated that equimolar CIDoMi primers had a wide SRF distribution, resulting in 5/11 genomes with >98% genome coverage. Although no individual amplicons had a median SRF of <0.05, optimization of CIDoMi primer concentrations increased the median SRF from 0.83 to 0.91 and generated >98% coverage from 9/11 isolate dilutions (Fig. 1C).

### Setting thresholds for minimum genome coverage for variant detection and clustering

**Fig 2:**
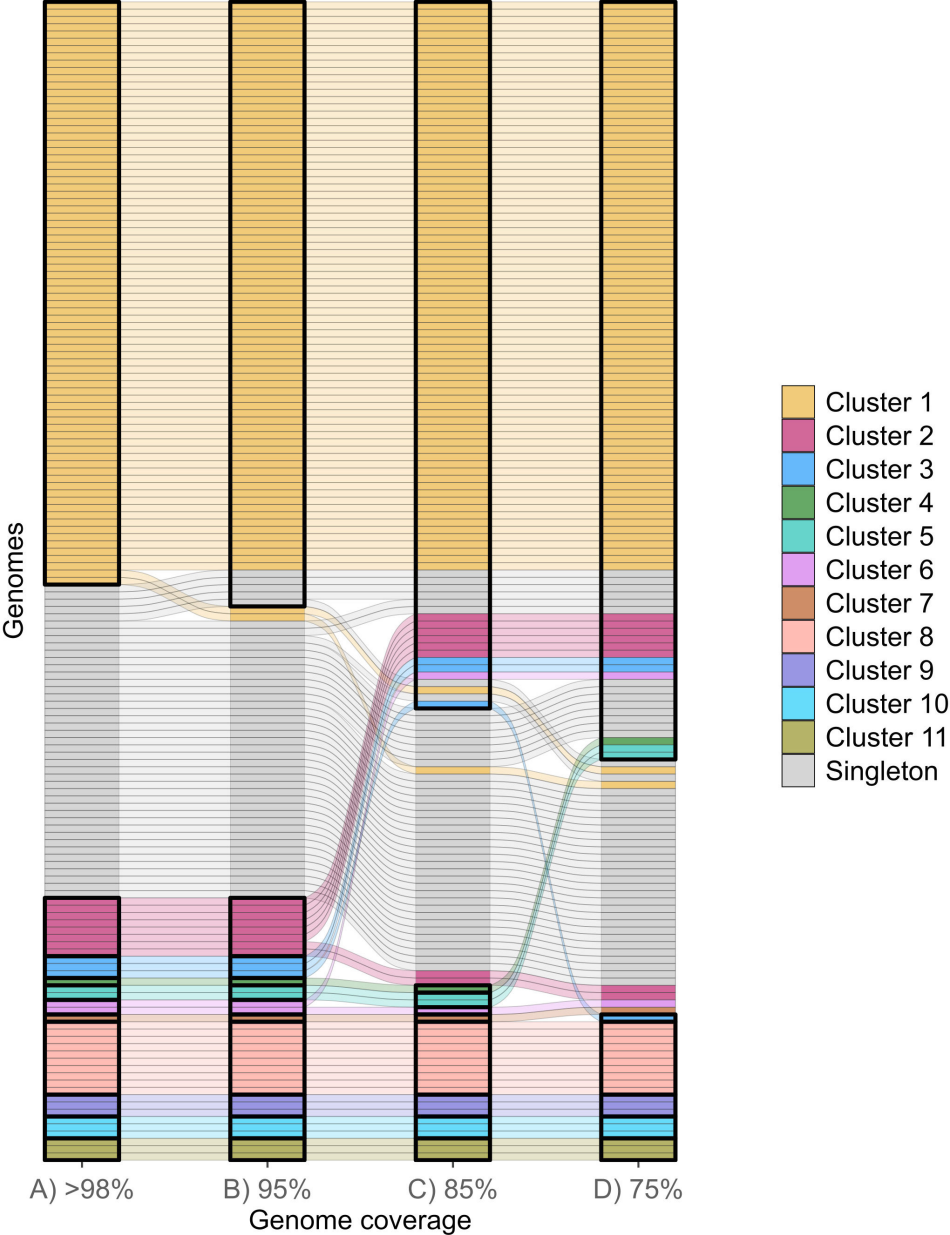
An alluvial plot demonstrating the changes in genomic clustering of 159 Delta variant of concern (VOC) samples as a result of simulated 5% (i), 15% (ii), and 25% (iii) reductions in genome coverage. Each horizonal bar within panels A to D represents a single Delta genome, which has been colored according to its cluster membership in panel A. Gray horizonal bars represent singleton Delta genomes that were not clustered. The colored and gray lines between columns trace the movement of individual samples into different genomic clusters as the level of genome coverage is reduced from panels A to D. Panel A depicts the true epidemiological and genomic clustering of all 159 genomes into 11 distinct clusters (depicted with vertical black boxes) when near-complete (>98%) genomes and comprehensive epidemiological data are available. Panels B, C, and D each represent genomic clustering with decreasing levels of genome coverage; (**B**) at 95% genome coverage, 11 genomic clusters are maintained, but Cluster 1 includes five additional epidemiologically unrelated singletons and excludes two epidemiologically linked cases; (**C**) at 85% genome coverage, only nine genomic clusters are defined with the loss of resolution of Cluster 2 and Cluster 3; and (D) at 75% genome coverage, only six genomic clusters are defined, with 18 cases known to be epidemiologically and genomically linked (at >98% coverage) that do not maintain the original cluster designation and 14 unlinked cases that join the largest genomic cluster (Cluster 1). The same set of 159 samples was used in each panel.

Genomic clustering was performed on a set of 159 SARS-CoV-2 Delta (B.1.617.2) genomes and found to be consistent with reported transmission events and epidemiological context (Fig. 2A). We estimated the effect of progressive loss of sequencing coverage on clustering capacity by masking critically underperforming amplicons (SRF < 0.05) from the genome in a stepwise manner. Three levels of genome coverage loss were simulated, corresponding to the loss of 5% (5 amplicons), 15% (15 amplicons), and 25% (25 amplicons) of the genome. At 95% genome coverage, regions of the genome corresponding to five amplicons with the lowest SRF were excluded during clustering analysis (amplicons 1, 5, 9, 11, and 18). At 85% genome coverage, genomic regions of an additional 10 amplicons (amplicons 7, 13, 10, 3, 60, 50, 33, 14, 23, and 88) were removed. The third simulation excluded a total of 25 amplicons to approximate 75% genome coverage (amplicons 29, 32, 64, 99, 44, 81, 26, 6, 77, and 42 in addition to the 15 amplicons mentioned above). At 95% genome coverage, the resolution of genomic clustering was reduced but was still sufficient to correctly assign the majority (94%, 150/159) of isolates into their original clusters or correctly identify them as singletons (gray bars) (Fig. 2B). Two isolates (1.3%, 2/159) that were previously in Cluster 1 could no longer be linked genomically, while five singletons (3%, 5/159) incorrectly joined Cluster 1. At 85% genome coverage (Fig. 2C), the ability to distinguish Cluster 2 and Cluster 3 was lost, as all three samples from Cluster 3 and six samples from Cluster 2 merged into Cluster 1; the remaining two samples from Cluster 2 became singletons. At 75% coverage, the genomic clustering appeared markedly different from the original high coverage (>98%) clustering (Fig. 2D). Only six clusters could be called with 75% genome coverage, compared with 11 clusters at >98% genome coverage. Furthermore, only four of these clusters (Clusters 8, 9, 10, and 11; Fig. 2D) retained the same samples as the original 98% coverage clustering. The loss of genome coverage also led to increasing heterogeneity in Cluster 1, which expanded to include 12 isolates from other clusters and 14 singletons. A further six isolates could not be genomically assigned to a cluster and became singletons (3.8%, 6/159). Notably, none of the singletons clustered with each other to form “pseudo-clusters” at any of the genome coverage levels.

### Monitoring genome sequencing coverage over time

We conducted a genome coverage review of SARS-CoV-2 genomes, which were generated as part of public health genomic surveillance of cases diagnosed in NSW between February 2020 and May 2022. Sequenced samples with >500 k mappable reads constituted 98.8% (23,606/23,893) of all SARS-CoV-2 genomes produced within our laboratory at the time and were expected to generate near-complete genomes with high depth of coverage. Only 1.2% (*n* = 287) of sequenced cases did not produce enough reads to be included in the retrospective genome coverage review.

**Fig 3:**
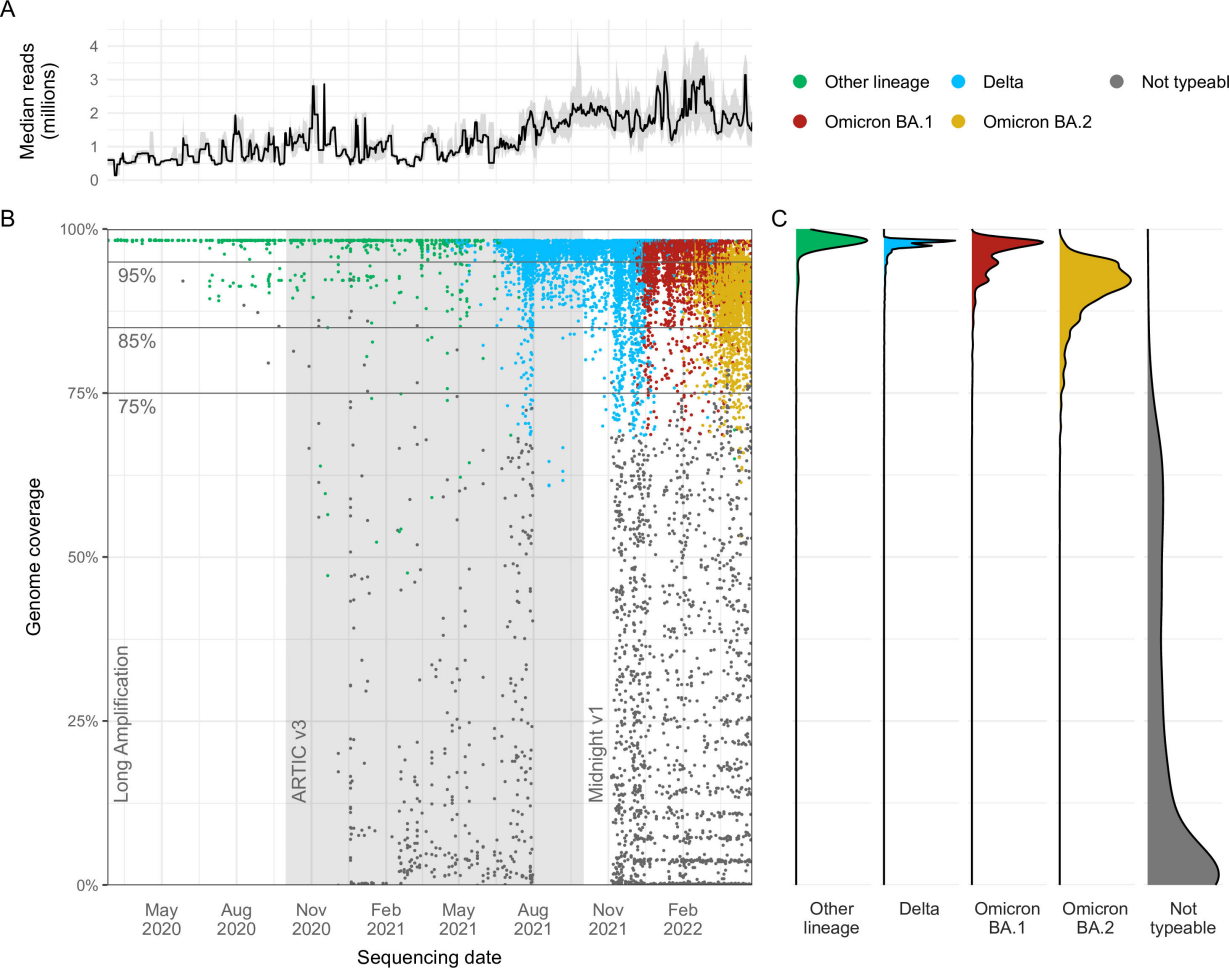
Genome coverage trends of 23,332 SARS-CoV-2 genomes collected in New South Wales (NSW) from January 2020 to 31 April 2022. (**A)** The upward trend in the median number of reads per genome over a rolling 1-week period; interquartile ranges of reads are shaded in gray. (**B)** Each colored dot corresponds to a genome designated as variants of concern (VOCs): Delta (blue), Omicron BA.1 (red) or Omicron BA.2 (yellow), or not typeable (gray). “Other lineage” (green) includes wild-type (Wuhan-like), Alpha, Beta, and Gamma variants. Changes in sequencing protocols over time (Long Amplification, ARTIC v3, and Midnight v1) are labeled in the background of the plot, while the distribution of percent genome coverage for each VOC category is shown in panel C.

Between January 2020 and August 2020, the average genome coverage was 98% using the long amplification protocol (5, 15), which gradually decreased to 95.7% in September 2020 (Fig. 3B). ARTIC v3 was introduced in October 2020 to replace the long amplification protocol and to improve the sensitivity of our genomic surveillance program as previously described. However, ARTIC v3 introduction was associated with a further drop in genome coverage to 91.4% by November 2020. Following the widespread co-circulation of Alpha and Beta in April 2021, genome coverage further declined to 75.1%. In October 2021, the Midnight v1 amplification protocol was implemented to better capture Delta genomes and led to an increase in average genome coverage to 96.3%; however, subsequent changes in the viral population and the emergence of Omicron (and associated sub-lineages BA.1 and BA.2) resulted again in a rapid reduction in genome coverage. Although the Midnight v1 amplification protocol performed well with Delta genomes, the average genome coverage for Omicron strains between December 2021 and April 2022 was only 83.6%.

We compared the distribution of genome coverage of the Delta, Omicron, and non-typeable genomes to determine overall trends in sequencing efficiency per SARS-CoV-2 variant category (Fig. 3C). The median genome coverage was 97.6% [interquartile range (IQR): 92.6%–98.2%] for Delta SARS-CoV-2, including wild-type, Alpha, Beta, and Gamma VOCs (“Other lineage”), 97.5% (IQR: 96.9%–98.3%) for Delta, 96.9% (93.9%–98.1%) for Omicron BA.1 and 91.7% (88.8%–98.7%) for Omicron BA.2, and 11.4% (0.3%–41.9%) for genomes that were not typeable.

A rolling 7-day median read count per genome is shown in Fig. 3A. The increase in the number of reads produced per genome can be seen as a gradual trend as each successive was introduced into NSW. During the initial phase of the COVID-19 pandemic when there was circulation of wild-type SARS-CoV-2, a median of 7.04 × 10^5^ reads was produced for each genome, which increased to 8.12 × 10^5^ and 1.00 × 10^6^ reads when Beta and Alpha began to co-circulate. The introduction of the Delta and corresponding investment in high-depth sequencing resulted in a rapid increase to 1.78 × 10^6^ reads per Delta genome. A further increase, representing a tripling of the number of reads per genome compared to the start of the COVID-19 pandemic, was observed with Omicron BA.1 (2.11 × 10^6^ reads) and Omicron BA.2 (1.98 × 10^6^ reads).

### Sequencing efficiency optimization framework for genomic surveillance

**Fig 4:**
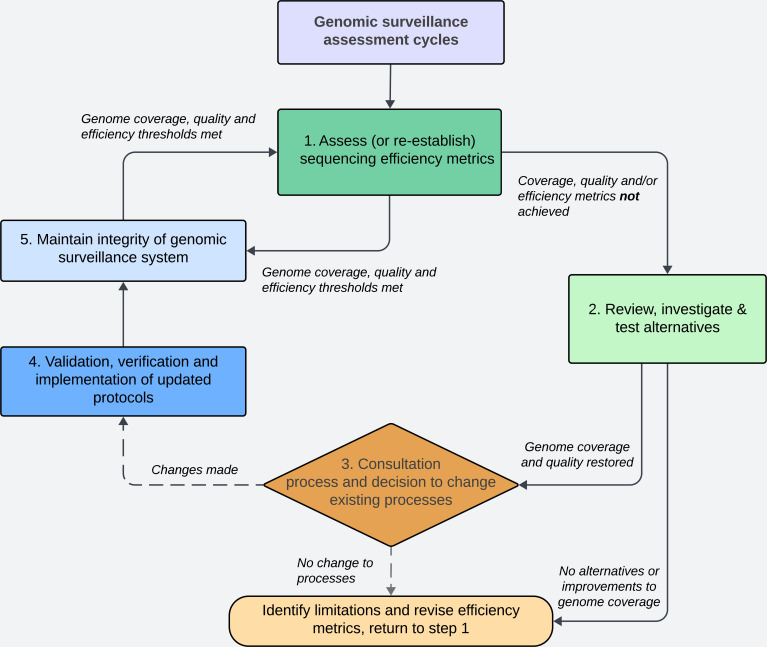
A quality framework to maintain and ensure high-quality genomic surveillance of viral pathogens. The framework represents an ongoing iterative process to maintain quality control within a genomic surveillance system, particularly when utilizing amplification-based whole genome sequencing (WGS). Each box represents an action that needs to be taken following the initial establishment of amplification-based viral WGS, while the diamond indicates a decision that needs to be made after considering the outcomes of the WGS review, as well as any results from testing alternative WGS methods. Arrows show the direction between one action and the next and is accompanied by a description of potential outcomes associated with each action.

We established a framework (Fig. 4) for monitoring and maintaining high-quality genomes to provide adequate resolution for different levels of genomic surveillance. The five-step framework was informed by ongoing optimization of SARS-CoV-2 amplification-based WGS methods employed at different times within our laboratory together with ongoing interrogation of the performance of these methods using routinely collected WGS metrics. Furthermore, the retrospective genome coverage review and simulation of genomic losses on surveillance resolution described in the results above provide additional evidence to support the quality control parameters established.

The first step of the framework, after the initial establishment of WGS surveillance, is careful and ongoing monitoring of sequencing metrics collected during routine WGS. Using these metrics, appropriate benchmarks for genome coverage were set and reviewed periodically. Three levels of genome coverage (>98%, 95%, and 85% genome coverage) were considered and corresponded to different levels of interpretation when used in genomic surveillance. When these benchmarks were not met, the second step of the framework was initiated. After successfully validating and implementing the ARTIC v3 protocol into routine SARS-CoV-2 WGS in October 2020, both gradual and sudden decreases in genome coverage below the lowest acceptable threshold of 85% were observed at different time points. Processes were then initiated to investigate reasons for poor sequencing performance (Fig. 4, Step 2).

In November 2020, a substantial proportion of sequences (6.67%) could not be typed; in the subsequent five months from November 2020 to April 2021, the average genome coverage had fallen to 75% in large part due to mutational changes in Alpha and Beta variants. The review process revealed that the accumulation of mutations, particularly within the spike region, had resulted in the failure to amplify key regions of the SARS-CoV-2 genome. As this phenomenon was occurring at a global level, the ARTIC Consortium released ARTIC v4 primers in June 2021 (22), which had been redesigned to accommodate high-frequency mutations arising from Alpha, Beta, Gamma, and Delta. Other amplification-based WGS protocols such as the Midnight protocol (4) had become available as alternative options. The 1,200-bp tiled PCR protocol had been designed with the same principles as ARTIC but with longer amplicons to accommodate long-read sequencing technology, allowing it to fit into existing laboratory processes with negligible impact on the sequencing workflow.

As part of the framework (Fig. 4, Step 2), we proceeded to test, optimize, and validate both methods against a selection of representative genomes [including the original wild-type SARS-CoV-2 (Lineage A.2.2), Beta, and Delta] to compare the performance of each method (results described above). The results of each of the WGS methods, as well as other factors (including the ability of each method to perform at scale, amount of verification and validation, and potential disruption to existing laboratory workflows), were used in the decision-making process (Fig. 4, Step 3) of whether to change existing workflows. In October 2021, routine SARS-CoV-2 WGS transitioned to using Midnight v1, and additional validation and verification processes were performed as part of the routine public health genomic surveillance service at our laboratory.

Each step of the framework was further refined when another iteration of decrease in genome coverage was observed. Substantial genomic changes within the viral population were again affecting the performance of the optimized sequencing protocol. Reports of sequences with a set of unusual mutations (23) and the first case of the SARS-CoV-2 Lineage B.1.1.529-designated Omicron had been identified in NSW on 27 November 2021 (24). While investigating alternative WGS methods or improvements to the Midnight v1 protocol, higher-depth sequencing was re-instated in order to sustain high-resolution local tracking of cases infected with Omicron. An update for ARTIC v4 was released in December 2021 involving the addition of 11 new primers (22), which also offered the potential to recover lost genome resolution. However, if implemented, the updated primers would still require optimization and rebalancing as well as updates due to the number of primers in the protocol and continuous representative surveillance. Given the characteristics and mutational profile of Omicron lineages, we opted to design the CIDoMi protocol, which provided us with over 99% of the genome sequence in the circulating Omicron genomes.

## DISCUSSION

The integration of high-resolution genomic surveillance into the public health response in NSW enabled the following applications of SARS-CoV-2 genomic data: (i) near-real-time clustering of cases and uncovering cryptic transmission events to support outbreak response and public health investigations, (ii) lineage assignment, and (iii) detection and identification of new and emerging variants. Such integration of genomic surveillance into a pandemic response improved the understanding of local transmission dynamics and public health outcomes (19, 25). This study describes cycles of the continuous optimization of sequencing efficiency, which has been formalized in a quality framework (Fig. 4) and has been essential for ensuring agility and sustainability of sufficient resolution for effective SARS-CoV-2 surveillance. Through iterative reviews and comparison of differences in genome coverage and SRF, we identified and investigated inefficient amplicons. Without optimization, inefficient amplicons can cause uneven distribution of reads across the genomes; as a result, informative SNPs may not be captured in poorly amplifying regions, particularly when sequenced at lower depths.

The impact of viral divergence on amplicon sequencing performance was clearly demonstrated by a sharp reduction in genome sequencing coverage associated with the emergence of successive SARS-CoV-2 VOCs. The accumulation of genomic differences from wild-type SARS-CoV-2 to Alpha, from Alpha to Delta, and particularly from Delta to Omicron was indicative of saltational evolution within the viral population (26) and likely driven by persistent infections within susceptible immunocompromised hosts (27). Saltational events (i.e., large multi-mutational jumps resulting in distinct and distant variants) have been predominantly observed within the N-terminal domain and receptor-binding domain of the spike protein and were also considered a feature of emergent SARS-CoV-2 VOCs (28). An unexplained reduction in genome coverage and clustering sensitivity can be treated as a signal of significant viral evolution, impacting the performance of sequencing protocols, once other explanations, such as laboratory contamination or index switching, are ruled out. Maintaining high-quality genomic surveillance is thus crucial in detecting these events to provide accurate genomic information for the development of diagnostic assays targeting these regions.

Based on our findings, a minimum 95% genome coverage threshold was established as a requirement for high-resolution genomic clustering of SARS-CoV-2, identification of cryptic transmission and disease transmission events, and early detection of variants. Reductions in clustering resolution were immediately apparent at a genome coverage of 95% but were still able to correctly identify the majority (94%) of epidemiologically linked clusters. Adequate quality genomes with coverage between 85% and 95% may still allow for genomic and epidemiological clustering if lineage-defining SNPs reside within high-performing amplicons. Lineage calling for these genomes may still also be feasible, which can then further guide the prioritization of samples for enhanced investigation. Our curated set of Delta genomes demonstrated that at 85% genome coverage, genomic links between related isolates were unable to be made, and missing genomic markers contributed to incorrect clustering. Clustering of unrelated cases (singletons) or merging or misassignment of defined genomic clusters can have significant misleading effects on follow-up public health actions. Our findings benchmarked the genome quality and coverage thresholds with a clear understanding of where incomplete genomic information can still be of value in a public health genomic surveillance system.

The decision-making process in this framework (Fig. 4, Step 3) is a key step and should be informed by available alternative processes identified during the review and investigation stages of this framework (Fig. 4, Step 2). Importantly, any decisions should only be made after consideration and consultation with relevant stakeholders who ultimately act upon the genomic data generated, including public health units and clinicians. For example, high genome resolution may be required when investigating cases of treatment failure to inform the best antiviral treatment strategy or for lineage designation of emergent variants. The benefits and limitations of changes to be made must also be conveyed during such assessment, as other key metrics may be affected, such as turnaround time for sequencing. The ability to produce and maintain high-quality genomes for laboratory surveillance depends on a range of (external) factors, including the viral yield within a sample and the speed of emergence of new variants. Pre-analytical factors such as sample collection, transport, and processing may interfere with the integrity of the original specimen and reduce the likelihood of recovering a complete genome (29). While such external factors are difficult to control, internal factors, such as sequencing platforms and equipment and competencies of laboratory and bioinformatic personnel, can be modified within an organization and are crucial factors in maintaining the integrity of the genomic surveillance system (Fig. 4, Step 5). An important consideration at the laboratory level for ongoing genomic surveillance is the implementation and validation of new or modified assays (Fig. 4, Step 4). We recommend the curation of a validation panel that can be used to retrospectively ensure that new amplification panels are able to detect existing variants of concern. Well-curated validation panels should be representative of circulating and historic variants of concern and, importantly, serve both wet and dry laboratory validation and verification processes.

In conclusion, the SARS-CoV-2 genome coverage and SRF can serve as informative indicators of reduction in performance due to viral evolution and act as triggers for sequencing protocol modifications. The proposed framework can help laboratories sustain high-resolution amplification-based public health genomic surveillance through prolonged epidemics of rapidly evolving pathogens and evolving public health containment approaches. In the context of increasing demand and associated costs of generating pathogen genomes, this sequencing optimization approach ensures the ongoing utility and accuracy of viral genomic surveillance programs. The COVID-19 pandemic informed the development of this framework, which can be further adapted to be pathogen agnostic and universally applied to other viral pathogens with epidemic potential.

## SUPPLEMENTAL MATERIAL

Fig. S1 spectrum.02202-23-s0001.eps Real-time PCR cycle threshold (Ct) values of culture extract dilutions.

Fig. S2 spectrum.02202-23-s0002.eps Box plot of sequence read fraction (SRF) values using Midnight, CIDoMi (original), and CIDoMi (rebalanced) primer sets.

Supplemental text spectrum.02202-23-s0003.docx Sequence data for design of CIDoMi SARS-CoV-2 WGS and retrospective review of SARS-CoV-2 sequences in NSW.

Supplemental tables spectrum.02202-23-s0004.xlsx Tables S1 to S5.

ASM does not own the copyrights to Supplemental Material that may be linked to, or accessed through, an article. The authors have granted ASM a non-exclusive, world-wide license to publish the Supplemental Material files. Please contact the corresponding author directly for reuse.
