# Supplementary material for: A laboratory framework for ongoing optimization of amplification-based genomic surveillance programs
Source: Microbiol Spectr. 2023 Nov 15;11(6):e02202-23. doi: 10.1128/spectrum.02202-23 (PMC10715188; doi:10.1128/spectrum.02202-23)
Supplement: Supplemental text — Sequence data for design of CIDoMi SARS-CoV-2 WGS and retrospective review of SARS-CoV-2 sequences in NSW. [file spectrum.02202-23-s0003.docx]

**Supplementary Data**

**Design of new SARS-CoV-2 whole genome sequencing protocol CIDoMI**

The seven SARS-CoV-2 genomes representing the following variants of concern: Delta AY39.1.2, Delta AY39.1.3, Omicron BA.1, BA.1.1, BA.1.17 and BA.2 were used as input into PrimalScheme to design CIDoMI primers and are available in the GISAID EPI_SET: [**10.55876/gis8.231006er**](https://doi.org/10.55876/gis8.231006er)**.**

Primer sequences and pooling volumes for the CIDoMI SARS-CoV-2 whole genome sequencing protocol is available in Supplementary Excel file.

**Retrospective review of SARS-CoV-2 sequences**

A total of 23,606 unique sequences were used for the retrospective review of SARS-CoV-2 whole genome sequencing between February 2020 and May 2022. Of these, 17,396 sequences met the upload criteria to GISAID and the sequence data for these samples are available in the GISAID EPI_SET**:** [**10.55876/gis8.230502km**](https://doi.org/10.55876/gis8.230502km)

Sequences which did not meet quality control criteria, duplicate sequences and non-typeable sequences have been excluded from the GISAID EPI_SET.

**Supplementary Tables**

**Supplementary Table S1:**

Pooling volumes for rebalanced ARTIC v3 primers

**Supplementary Table S2:**

Pooling volumes for rebalanced ARTIC v4 primers

**Supplementary Table S3:**

Pooling volumes for rebalanced Midnight v1 primers

**Supplementary Table S4:**

Pooling volumes for rebalanced CIDoMI primers

**Supplementary Table S5:**

Primer sequences for CIDoMI SARS-CoV-2 whole genome sequencing

**Supplementary Figure legends**

**Supplementary Figure 1:**

Real-time PCR cycle threshold (Ct) values of culture extract dilutions in this study. Serial dilutions of SARS-CoV-2 cultures corresponding to Lineage A.2.2.2 (yellow), Variants of Concern Beta (green), Delta (teal), Omicron BA.1 (pink), BA.2 (blue). The average SARS-CoV-2 Ct value of all complete genomes was 29.55 (range 25.58-33.94) compared to 35.93 for incomplete genomes (range 32.85 - 38.22).

**Supplementary Figure 2:**

Boxplot demonstrating the range of sequence read fractions (SRF) across the SARS-CoV-2 genome using A) Midnight, B) CIDoMI (original), and C) CIDoMI (rebalanced) primer set. The red dashed line indicates the SRF threshold (<0.05) for critically low performing amplicons. Consistently poor performing amplicons in the Midnight panel included amplicon 24 (within the Spike region) and amplicon 28 in the N-gene, with both SRF <0.05, even when rebalanced. Both regions were recovered in the CIDoMI (original) and CIDoMI (rebalanced) primer sets, with overall higher SRFs for the CIDoMI (rebalanced) panel and no critically underperforming amplicons.
